# An Assessment of Personal Hygiene Practices Among Young Adults: A Cross-Sectional, Descriptive Study

**DOI:** 10.7759/cureus.44308

**Published:** 2023-08-29

**Authors:** Pramod Singh, Abdul Rafae Faisal, Mohammad Maaz Alam, Ahmad Saeed, Tauqeer Haider, Hafiz Muhammad Awais Asif, Jeevan Rauniyar, Niraj Rai, Muhammad Asad Khan Janjua, Abdul Rafay Pasha

**Affiliations:** 1 Nephrology, Punjab Medical College, Faisalabad Medical University, Faisalabad, PAK; 2 Nephrology Department, Faisalabad Medical University, Faisalabad, PAK; 3 Medicine, Allied Hospital, Faisalabad, PAK; 4 Medicine, Primary Health Care (PHC) Bhojpur, Bhojpur, NPL; 5 Psychiatry, Punjab Medical College, Faisalabad Medical University, Faisalabad, PAK; 6 Medicine, Holy Family Hospital, Rawalpindi, PAK; 7 Department of Psychiatry, Allama Iqbal Medical College, Lahore, PAK

**Keywords:** prevention, health promotion, behavior, practices, personal hygiene

## Abstract

Background

Personal Hygiene behavior and practices play a major role in health promotion and disease prevention. Socio-demographic, behavioral, and psychological factors sway a person’s overall adaptation of good practices. Disease burden leading to loss of productivity and its influence on the economy cannot be overstated. As medical professionals come in contact with a large number of people, they carry the potential to be super-spreaders in disease outbreaks. Thus, it is of utmost importance that medics and paramedics maintain hygiene to the highest standards.

Method

This cross-sectional study comprised 323 young adult participants from Muslim Town, Faisalabad. A structured questionnaire containing close-ended questions was used for data collection regarding personal hygiene behavior and practices. Dependency between various qualitative categorical variables and hygiene practices was analyzed using Chi-squared tests.

Results

It was found that although the majority of the participants followed good hygiene for most of the practices; laziness, lack of relevant education, improper time management, and unreliable water supply posed a significant barrier to good practices. Females had demonstrably better practices compared to males. Urbanity did not have a significant correlation with the results.

Conclusion

We conclude that a rigorous program of awareness and education regarding this subject is the need of the hour to facilitate an improvement in predictive and preventive health care and reduce morbidity and mortality. Steps should be taken to ease the barriers that are obstructing optimal hygienic practices.

## Introduction

The UN’s Sustainable Development Goal of good health and well-being has been recognized globally owing to its aim of reducing mortality [1]. The achievement of this goal depends upon the substitution of traditional and customary practices with an approach towards preventive medicine. The journey towards innovative and advanced health care has opened new vistas for medical treatment but it has also allowed a gradual shift from delayed interventions to predictive medicine that is suited to the person’s needs. There is an increasing role of patient participation. The trend now is from reactive to preventive medicine [2-4]. Thus, Predictive Preventive and Personalized Medicine (PPPM) is starting to occupy a central position in efforts in health care aimed at minimizing the incidence of both communicable and non-communicable diseases in global communities [5].

PPPM is a fresh and all-encompassing concept in health care that enables the prediction of risk before disease onset. It serves to provide targeted measures for prevention and creates personalized treatment options and plans [2,5]. The expected outcomes help in more effective population screening, identifying people at risk and reducing adverse impacts on health [4]. Personal hygiene practices are at the forefront of PPPM and are indispensable in its successful application. That is even more true of developing countries with poorer populations and insufficient sanitary facilities. Personal hygiene plays a big part in disease prevention and health promotion [6-8]. Hygienic practices are strongly influenced by social, familial and individual factors, as well as the individual’s knowledge and attitudes towards hygiene [9,10]. It is correlated with an individual's background, education, and upbringing.

As our world contracts into a global village of sorts, personal hygiene practices hold even greater importance in the public health sphere regarding the spread of easily transmissible infections that could lead to a worldwide pandemic. That is becoming abundantly clear in the context of the coronavirus disease 2019 (COVID-19) pandemic crisis that is ravaging nearly every country. The only reliable way to slow and stop its spread is a firm adherence to the principles of personal hygiene [11]. Hence, the maintenance of high standards of hygiene helps to prevent the outbreak and spread of infections as well [12]. The most important part of any preventive measure must include an analysis of the behavior of a given population. The personal hygiene habits practiced by a given population can provide a reliable estimation for the successful application of the predictive and preventive medicine process.

In any healthcare and delivery system, doctors and healthcare workers play the most important role. They can serve as role models and set an example for others to follow. Hence, the assessment and improvement of their personal hygiene practices hold undeniable significance. The maintenance of hygiene and the requisition of sanitation have a direct and potent impact on economic growth and development. Poor hygiene and sanitation result in economic losses tied directly to the treatment costs of sanitation-related diseases and a loss in prospective income due to reduced productivity. Even in the 21st Century, improper hygiene and insufficient sanitation account for the heaviest loads of disease burden globally [13,14]

Poor adherence to personal hygiene principles causes, in part, 4% of all deaths and 5.7% of disability worldwide [15]. It stunts and debilitates economic progress and development. The prevention of infectious diseases has become one of the biggest challenges facing developing countries all over the world, including Pakistan. It has over-stretched the already meager resources of these countries which often have poorly structured and under-funded health care systems. It also puts a tremendous strain on the economies of such countries and further cripples their economic productivity. Considering the importance of this study and the issue regarding personal hygiene, this study will help the health authorities in controlling and spreading awareness.

## Materials and methods

Study location, design, and sample size

The study was conducted in Muslim Town, Faisalabad. A cross-sectional study design was employed for the study. A sample size of 323 participants was used. A structured questionnaire containing close-ended questions was used for data collection regarding personal hygiene behavior and practices. The study population comprised young adults (individuals between ages 15-29 years) [16] of Muslim Town, Faisalabad (Urban Area).

The minimum sample size (n) was calculated as 323 with an estimated population (N) of 2000 with a confidence level set at 95% with an allowable margin of error of 5%. The baseline level of indicators is 0.5. The z-score was determined to be 1.96 at a 95% confidence interval with the help of z distribution table. The estimated population proportion was fixed at 0.5 in the absence of similar research on the population. The following formula [17] was used for the determination of sample size.

$n=((z^2 &times;p (1-p))/e^2 )/(1+(z^2 &times;p (1-p))/(e^2 N))$

N = population size; e = margin of error; z = Z score; p = estimated population proportion

Sampling technique

Non-random convenience sampling was utilized. All the young adults of Muslim Town, Faisalabad (Urban Area) were considered for sampling.

Data collection and analysis

The study took place in the month of March 2020. A structured close-ended questionnaire was used for collecting categorical and quantitative data of participants. The survey comprised 24 questions in total divided into three specific sections enquiring about socio-demographic details, personal hygiene practices, and barriers to personal hygiene respectively. The socio-demographic section revealed information about Age, Sex, Residence, Boarding status, Father’s educational status, and Academic qualification of the participants. Questions pertaining to hygiene practices scored participants' responses on oral hygiene, nail hygiene, bathing hygiene, hair care, underwear hygiene, and hand washing. The next section collected the views of the respondents about what they thought as barriers to their personal hygiene.

Each of the questions relating to personal hygiene practices used a common scoring system for the quantification of collected data. Three possible responses were made available and weighted as none = 0 (poor practice), sometimes = 1 (moderate practice), and regularly =2 (good practice). For the purpose of data description, mean (x) and standard deviation (SD) were calculated. Percentile scores of mean were used for characterizing overall hygiene practice, with those having their mean hygiene score below the 25th percentile characterized as Poor Practice (PP), 25th and 75th percentile as Moderate practice, and above 75th percentile as Good practice. Differences among categorical variables such as age, gender, and educational status were also analyzed using the same scoring system.

Ethical consideration

The expressed consent of each participant was taken before data collection. The participants were properly informed about the purpose of the study. They were also made aware that no personal identifier would be used in any shape or form, assuring them of their confidentiality status. Institutional Ethical Review Committee, Faisalabad Medical University, Faisalabad, approved the study (Registration No. 1038).

Data handling and analysis

Data was entered into a spreadsheet in Microsoft Excel v 2016. The spreadsheet was later imported into R v.3.6.3 (R Foundation for Statistical Computing, Vienna, Austria) for data visualization and descriptive and inferential analysis. Proportions, Means, and Standard deviations were used for comparative study between various socio-demographics. Contingency tables were created and subsequently analyzed using Chi-squared (X2) tests for assessing dependency between multiple qualitative categorical variables and hygiene practice. Hypothesis testing was done by implementing the null hypothesis that various socio-demographic variables are mutually independent of one another. Consequently, the alternate hypothesis of dependency was analyzed using p-values obtained through Pearson’s Chi-squared test.

## Results

Table 1 describes the frequency distribution of various socio-demographic characteristics of the sample. Most of the respondents belonged to the 19-25 age group (87.41%). Age group 26 and above was the least (2.22%) represented demographic. There were more males (59.01%) than females (40.98%) in the sample. In addition, urban residents (78.27%) constituted the majority while only 21.73% were of rural residence. Moreover, the minority of the respondent’s fathers (2.22%) were illiterate, while the majority (78.77%) had studied up to intermediate level or above.

**Table 1:** Sociodemographic characteristics of respondents

Socio-demographic variable	Frequency(N)	Percentage (%)
Age		
18 and below	42	10.37
19-25	354	87.41
26 and above	9	2.22
Sex		
Female	166	40.98
Male	239	59.01
Residence		
Rural	88	21.73
Urban	317	78.27
Father’s Educational Status		
Illiterate	9	2.22
Primary or below	11	2.71
Secondary or below	66	16.30
Intermediate or above	319	78.77
Total	405	

Table 2 is a description of respondent’s hygiene practices regarding specific questions that were asked in the questionnaire. Among the sample, practice regarding the use of lavatory is the most hygienic with 93.8% employing good practice, 4.2% having moderate practice, and only 2% undertaking bad practice. Likewise, 88.9% followed good hygiene practices when it came to brushing their teeth and wearing washed clothes regularly. On the other hand, only 45.7% washed their hands regularly before touching their privates with 22% responding that they never did. Similarly, only 52.1% responded that they followed good hygiene practices while picking their nose.

**Table 2:** Hygiene practices of respondents

Description	Good Practice No(percentage)	Moderate Practice No(percentage)	Bad Practice No(percentage)	
How often do you brush your teeth	360(88.9%)	39(9.6%)	6(1.5%)	
How often do you cut your nails?	273(67.4%)	125(30.9%)	7(1.7%)	
How often do you wear washed clothes?	360(88.9%)	38(9.4%)	7(1.7%)	
How often do you change your underwear?	306(75.6%)	84(20.7%)	15(3.7%)	
How often do you remove unwanted hair?	292(72.1%)	107(26.4%))	6(1.5%)	
How often do you wash your hair?	316(78.0%)	85(21.0%)	4(1%)	
How often do you use a handkerchief/ tissue while picking your nose?	211(52.1%))	165(40.7%)	29(7.2%)	
Do you wash your hands before eating?	294(72.6%)	102(25.2%)	9(2.2%)	
Do you wash your hands with soap after using the toilet?	380(93.8%)	17(4.2%)	8(2%)	
Do you wash your hands after wiping or blowing your nose?	245(60.5%)	140(34.6%)	20(4.9%)	
Do you wash your hands after touching animals?	321(79.3%)	69(17.0%)	15(3.7%)	
Do you wash your hands before touching your privates?	185(45.7%)	131(32.4%)	89(22.0%)	
Do you wash your hands after touching your privates?	331(81.7%)	58(14.3%)	16(4%)	

Table 3 portrays information about the barriers to respondents’ hygiene practices. 94.6% (383) said that laziness was a major factor in exercising their hygiene practice. This was followed by a lack of proper education, water supply, and inadequate time, with 94.6%, 81.9%, and 55.4% replying in the affirmative. Religion acted as a barrier to hygiene for only 27.1% of the respondents.

**Table 3:** Barriers to personal hygiene

		Barriers to Personal Hygiene					
Variable			Male		Female		Total
		N	%	N	%	N	%
Lack of education		192	80.3%	157	94.6%	349	86.2%
Lack of water supply		174	72.8%	136	81.9%	310	76.5%
Lack of time		119	49.8%	92	55.4%	211	52.1%
Religious beliefs		70	29.3%	45	27.1%	115	28.4%
Laziness		222	92.9%	161	97.0%	383	94.6%

**Table 4:** Mutual dependency test (Pearson’s Chi-squared test)

	Mutual dependency test (Chi-Squared Test) between Hygiene Practices and Socio-demographic variables												
Variables		Good Practice			Moderate Practice			Poor Practice			Pearson's Chi Squared Test		
		N	%		N	%		N	%		P value, X^2^		
Age													
18 and below	4		9.5		27	64.3		11	26.2		X^2^= 9.9015 p-value = 0.0421		
19-25		54	15.3		216	61		84	23.7				
26 and above	0		0		3	33.3		6	66.7				
Gender													
Male		36	15.0		128	53.6		75	31.4		X^2^= 14.884 p-value = 0.0005		
Female		22	13.3		118	71.1		26	15.6				
Residence													
Rural		17	19.3		49	55.7		22	25		X^2^ = 2.4343 p-value = 0.2961		
Urban		41	12.9		197	62.1		79	24.9				
Father's literacy													
Illiterate		3	33.3		3	33.3		3	33.3				
Primary or below	3		27.3		5	45.4		3	27.3		X^2^ = 6.2142 p-value = 0.3996		
Secondary or below	10		15.1		42	63.6		14	21.2				
Intermediate or above	42		13.2		196	61.4		81	25.4		X^2 ^= 21.313 p-value = 0.2639		
Mutually Dependent if P-value < 0.05													

Table 4 shows the results of Pearson’s Chi-squared test (X2) between various socio-demographics and their hygiene practices. The significance testing is demonstrated by the p-value with p-value < 0.05 implying that the observed effect was significant and there is mutual dependency. From the table, it can be observed that age was a significant factor in hygiene practice (p = 0.0421). Age group 19-25 had significant good practice at 15.3% with 61% reporting moderate practice. On the other hand, the age group 26 and above had 66.7% reporting bad practice. Moreover, females outperformed males in hygiene practices. 84.4 % of females had either good or moderate practice, while only 68.6% of males had similar hygiene practices (p=0.0005). In contrast, the testing did not show a significant dependency between rural and urban residency with hygiene practice (p=0.2961). Similarly, neither Father's literacy level (p=0.3996), nor the academic program of study (p=0.2639) showed a significant relationship with hygiene practice. However, it was also observed that there was a significant dependency between residential status and hygiene practice.

## Discussion

Poor health practices and sanitation are associated with a myriad of serious medical conditions including diarrhea, cholera, dysentery, polio, hepatitis, and worm infestations. These diseases are all easily preventable by adopting basic hygiene practices and providing basic sanitation amenities [18].

The assessment of hygiene behavior is an essential component of the equation for the generation of accurate estimates in populations regarding disease occurrence and also forms the backbone of preventive and predictive medicine [19-21]. It allows us to assess the attitude of the population regarding individual health and judge their mental makeup and personal beliefs about this topic. These beliefs are informed and entrenched in societies and often are a product of a diverse multitude of factors. The ultimate objective of this paper is to evaluate the personal hygiene practices among university students of Faisalabad Medical University, Faisalabad, with the goal of making future efforts to improve targeted interventions for young people. With the young being the torch bearers of tomorrow, personal hygiene among the youth of today becomes ever more essential as it forms and informs the general well-being and health of the individual and society as well [22]. Personal hygiene practice is affected by many factors which are the developmental level, cultural background, socioeconomic status, personal habits, and health status [9,23]

In this study, we found that a majority of respondents engaged in good hygiene practices for all the described activities (Table 2). The practices (Table 2) that were generally strong among study participants included washing hands after using the toilet (93.8%), brushing teeth (88.9%), washing hands after touching their private parts (81.7%), and wearing washed clothes (88.9%). The high scores in brushing and hand-washing habits were consistent with previous studies [24-26].

Most of this study's participants belonged to the age group of 19-25 years. Amongst a cohort of 405 young adults, 354 individuals were categorized within this range, with 42 being under the age of 18 and a mere nine exceeding 26 years of age. Hence, most of the respondents have just passed through adolescence. It is expected of them to have learnt and applied the sound principles of personal hygiene. The study also explored (Table 4) the relationship between hygiene practices, age, gender, and residential status. It was observed that the practices deteriorated with age. Curiously, we noted that the female participants had better personal hygiene practices as compared to males. This is consistent with a multitude of previous studies [27,28]. The results showed an insignificant influence of urbanization on good hygiene behavior of the young adults of Muslim Town, Faisalabad, and was not a statistically significant factor, which is in contrast to previous studies [29].

The results from this study (Table 3) indicate that the most significant barrier to personal hygiene from the perspective of the respondents is laziness (94.6%) followed by lack of education (86.2%) and lack of time (76.5%). The fact that a large majority of people consider indolence and inertia as major barriers to adherence to the practices of personal hygiene is concerning. The lack of education and awareness regarding this subject also needs addressing on an urgent basis. The majority of the respondents were either adolescents or young adults. A considerable majority of the respondents were engaged in good hygiene practices in all the brackets, which was a significant improvement compared to past studies. Females had demonstrably better practices compared to males. Urbanity did not have a significant correlation with the results. We conclude that a rigorous program of awareness and education regarding this subject is the need of the hour to facilitate an improvement in predictive and preventive health care and reduce morbidity and mortality.

## Conclusions

We conclude that the importance of implementing a comprehensive program focused on raising awareness and educating individuals about a specific subject relates to predictive and preventive healthcare. This program is considered crucial for addressing issues related to morbidity and mortality (death) rates. This also highlights the necessity of overcoming obstacles that hinder optimal hygienic practices. Let's break down the statement and its key points: Rigorous Program of Awareness and Education, Improvement in Predictive and Preventive Healthcare, Reducing Morbidity and Mortality, and Ease Barriers to Optimal Hygienic Practices.

In summary, this advocates for the implementation of a comprehensive awareness and education program to enhance predictive and preventive healthcare, ultimately leading to a decrease in morbidity and mortality rates. Additionally, it emphasizes the importance of identifying and overcoming barriers that impede optimal hygienic practices. Such a program could involve a range of activities, such as workshops, public campaigns, online resources, community outreach, and partnerships with healthcare providers, to empower individuals with the knowledge and skills needed to make informed decisions about their health and well-being.
